# Serotonergic and dopaminergic control of impulsivity in gambling disorder

**DOI:** 10.1111/adb.13264

**Published:** 2023-01-10

**Authors:** Valtteri Kaasinen, Emma A. Honkanen, Kari Lindholm, Elina Jaakkola, Joonas Majuri, Riitta Parkkola, Tommi Noponen, Tero Vahlberg, Valerie Voon, Luke Clark, Juho Joutsa, Marko Seppänen

**Affiliations:** ^1^ Clinical Neurosciences, Department of Clinical Medicine, Faculty of Medicine University of Turku Turku Finland; ^2^ Neurocenter Turku University Hospital Turku Finland; ^3^ Turku PET Centre Turku University Hospital Turku Finland; ^4^ Department of Neurology North Kymi Hospital Kouvola Finland; ^5^ Department of Radiology University of Turku and Turku University Hospital Turku Finland; ^6^ Department of Clinical Physiology and Nuclear Medicine University of Turku and Turku University Hospital Turku Finland; ^7^ Department of Medical Physics Turku University Hospital Turku Finland; ^8^ Biostatistics, Department of Clinical Medicine, Faculty of Medicine University of Turku Turku Finland; ^9^ Department of Psychiatry University of Cambridge Cambridge UK; ^10^ Institute of Science and Technology for Brain‐Inspired Intelligence Fudan University Shanghai China; ^11^ Department of Psychology and Djavad Mowafaghian Centre for Brain Health University of British Columbia Vancouver British Columbia Canada; ^12^ Turku Brain and Mind Center, Department of Clinical Medicine, Faculty of Medicine University of Turku Turku Finland

**Keywords:** gambling disorder, impulsivity, SPECT

## Abstract

Gambling disorder (GD) is major public health issue. The disorder is often characterized by elevated impulsivity with evidence from analogous substance use disorders underlining prominent roles of brain monoamines in addiction susceptibility and outcome. Critically, GD allows the study of addiction mechanisms without the confounder of the effects of chronic substances. Here, we assessed the roles of striatal dopamine transporter binding and extrastriatal serotonin transporter binding in GD as a function of impulsivity using [^123^I]FP‐CIT SPECT imaging in 20 older adults with GD (DSM‐5 criteria; mean age 64 years) and 40 non‐GD age‐ and sex‐matched controls. We focused on GD in older individuals because there are prominent age‐related changes in neurotransmitter function and because there are no reported neuroimaging studies of GD in older adults. Volume‐of‐interest‐based and voxelwise analyses were performed. GD patients scored clearly higher on impulsivity and had higher tracer binding in the ventromedial prefrontal cortex than controls (*p* < 0.001), likely reflecting serotonin transporter activity. The binding in the medial prefrontal cortex positively correlated with impulsivity over the whole sample (*r* = 0.62, *p* < 0.001) as well as separately in GD patients (*r* = 0.46, *p* = 0.04) and controls (*r* = 0.52, *p* < 0.001). Striatal tracer binding, reflecting dopamine transporter activity was also positively correlated with impulsivity but showed no group differences. These findings highlight the role of prefrontal serotonergic function in GD and impulsivity. They identify cerebral coordinates of a potential target for neuromodulation for both GD and high impulsivity, a core phenotypic dimensional cognitive marker in addictions.

## Graphical abstract

The results demonstrate increased prefrontal serotonin transporter function in gambling disorder that is associated with individual differences in impulsivity. An implication of this is the possibility that pathologically increased impulsive behaviour may be modulated in humans by interventions that target serotonin function in the prefrontal cortex.

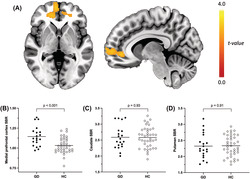

## 1 INTRODUCTION

Gambling disorder (GD), characterized by persistent, recurrent maladaptive patterns of gambling behaviour, is a public health concern worldwide.^1^ The increasing recognition of GD is combined with the limited success of current treatment approaches,^2^, ^3^ and there are no pharmacological agents approved in any country for the treatment of GD.^4^ A key task in the field is therefore the translation of the underlying neurobiological mechanisms into better prevention and treatment strategies for GD.^4^, ^5^

The neurobiology of GD remains largely unclear but brain circuits involving ventral prefrontal, ventral striatal and limbic brain regions seem to contribute to reward‐related decision‐making biases that drive uncontrolled gambling.^5^ Multiple neurotransmitter systems in these circuits have been implicated, but the brain monoamines dopamine (3,4‐dihydroxyphenethylamine, DA) and serotonin (5‐hydroxytryptamine, 5‐HT) play major roles in impulsive and compulsive behaviours. Studies of DA function in reward‐related processes have been instrumental in our understanding of compulsive drug‐seeking behaviour,^6^ but further adaptations in the 5‐HT system are also implicated in susceptibility to compulsive drug use.^7^ In healthy individuals, and with relevance to GD, the 5‐HT system seems to modulate decision‐making under risk.^8^ However, DA or 5‐HT neuroimaging studies in GD are scarce and have shown inconsistent results. For DA function, studies have reported no changes in DA D_2/3_ receptor binding in GD,^9^, ^10^, ^11^, ^12^, ^13^ increased striatal DA synthesis capacity,^14^ no change in DA synthesis capacity,^15^ and decreased striatal DA transporter (DAT) binding.^16^ The lack of data is even more notable for 5‐HT neuroimaging with only two reported studies in GD, one finding no evidence of alterations in 5‐HT 1B receptor binding in GD^17^ and the other reporting the same for 5‐HT transporter (SERT) binding.^18^ Moreover, samples in GD neuroimaging studies have generally been small (total number of GD Subjects *n* = 12–18),^19^ which could explain the inconsistencies and negative results.

Although GD is highly heterogeneous in terms of phenotypic characteristics, patients are characterized by high impulsivity across various subdomains of impulsivity,^20^ and impulsivity is also associated with psychiatric comorbidity (e.g., ADHD). In substance use disorders, increased impulsivity is a robust phenomenon across different addictions, is associated with clinical outcomes, and can be a pre‐existing risk for vulnerability and dependence.^21^ From a therapeutic point of view, there is increasing evidence that supports pharmacological and behavioural treatments for impulsivity in substance use disorders,^22^ but the underlying mechanisms and treatment targets are poorly defined, particularly for high impulsiveness in GD.

In the present study, we aimed to fill the knowledge gap concerning monoaminergic function in GD with a focus on impulsivity, a prominent abnormality in GD patients. For the neuroimaging tracer, we used [^123^I]FP‐CIT, a cocaine analogue that has high affinity for DAT in the striatum but also a moderate affinity for SERT outside the striatum, thus enabling a simultaneous investigation of two monoamine transporters in one imaging session.^23^, ^24^, ^25^ We focused on GD in older individuals because there are prominent age‐related changes in neurotransmitter function that may be relevant to pharmacological treatments and addictions^26^ and because there are no reported neuroimaging studies of GD in older adults. Our sample size represents the largest GD serotonergic or dopaminergic neuroimaging study to date.

## 2 MATERIALS AND METHODS

### 2.1 Subjects

Twenty individuals with GD and 40 non‐GD controls were recruited (Table 1). All subjects were Caucasian and of Finnish nationality. Subjects were included if they fulfilled DSM‐5 criteria for GD and were between ages 50–85 years. The sample size and 1:2 sampling ratio were based on power calculations and expected number of recruitable GD patients within the time span of the study. Elderly individuals were examined because of nearly complete lack of neurobiological studies focusing on older patients with GD and to provide comparative data for later studies with Parkinson's disease patients suffering from impulse control disorders. Subjects were excluded if they had serious neurological or other psychiatric disorders. Subjects were interviewed and examined using the same structured interview and criteria. Screened psychiatric problems were: behavioural addictions including GD, current alcohol or substance use disorder within the last 6 months (DSM‐5), ADHD, current other axis I disorders such as major depression, bipolar disorder or psychotic disorder, current treatment with amphetamine derivatives, methylphenidate, bupropion or other medications known to interfere with DAT imaging. In addition, possible psychiatric problems without diagnoses were inquired together with earlier (>6 months before the start of the study) regular use of tobacco, smokeless tobacco (snus), nicotine products or other addictive substances (alcohol, prescription drugs or illicit drugs). The subjects were recruited via media advertisements in community and the participants underwent a gambling behaviour interview including the South Oaks Gambling Screen (SOGS), which shows good internal consistency and a strong correlation with gambling symptoms,^27^ together with a clinical interview, the Unified Parkinson's Disease Rating Scale (MDS‐UPDRS) Part III for possible motor symptoms and signs, the Mini‐Mental State Examination (MMSE), the Beck Depression Inventory (BDI), the Beck Anxiety Inventory (BAI) and the Barratt Impulsiveness Scale (BIS‐11). All subjects were clinically examined 2–4 h before imaging. All participants gave written informed consent for participation in the study. Each subject received a monetary compensation of 120 € for participation. The study was approved by the local Ethics Committee and was conducted according to the Declaration of Helsinki at Turku University Hospital, Finland, in 2019–2020.

**TABLE 1:** Main demographic and clinical characteristics of the studied GD patients and controls. Values are *n*, mean (SD) or median [IQR]

Test domain	Test/variable	Gambling disorder	Healthy controls	*p*‐value
**Sample size**	*n*	20	40	‐
**Demographics**	Age (years)	64.0 (5.7)	66.8 (9.0)	0.14
	Sex (m/f)	12/8	21/19	0.78
	BMI (kg/m^2^)	30.1 [5.9]	26.8 [5.4]	0.01
	Handedness (r/l)	18/2	36/4	1.0
	Formal education (years)	12 [6]	15 [5]	0.02
**Alcohol and nicotine**	Alcohol (drinks/week)	2.5 [9]	2.0 [5]	0.57
	Currently smoking or using nicotine products (y/n)	5/15	3/37	0.10
**Motor function**	MDS‐UPDRS part III	8.5 [9]	5.0 [7]	0.06
	Hand grip strength, mean of left and right (kg)	36.4 (11.0)	36.7 (11.5)	0.93
**Cognition**	MMSE	28 [3]	28 [3]	0.50
**Depression**	BDI	4.5 [8]	0.5 [5]	0.007
**Anxiety**	BAI	6 [5.5]	3 [6]	0.06
**Impulsivity and Gambling**	BIS total score	66.8 (8.7)	57.4 (6.4)	<0.001
	BIS attentional	15.4 (3.2)	13.0 (2.3)	0.002
	BIS motor	24.2 (3.8)	21.6 (2.8)	0.005
	BIS nonplanning	27.3 (5.0)	22.8 (4.5)	<0.001
	Duration of problem gambling (years)	11 [24]	0 [0]	<0.001
	SOGS	9.2 (2.9)	0 [0]	<0.001
	Gambling per week (hrs)	7.3 [11]	0 [0]	<0.001
	Monetary loss per week (€)	110 [229]	0 [0]	<0.001
	Gambling debt (€)	825 [36500]	0 [0]	<0.001
**Tracer binding (SBR)**	Caudate right	2.54 (0.31)	2.62 (0.37)	0.41
	Caudate left	2.61 (0.36)	2.55 (0.41)	0.58
	Putamen right	2.35 (0.32)	2.33 (0.37)	0.80
	Putamen left	2.32 (0.35)	2.32 (0.43)	0.98
	Medial PFC	1.14 (0.12)	1.03 (0.10)	<0.001

*Note*: *p*‐values are from independent samples *t*‐test, Fisher's exact test or Mann–Whitney U‐test.

Abbreviations: BAI, Beck Anxiety Inventory; BDI, Beck Depression Inventory; BIS, Barratt Impulsiveness Scale; MMSE, Mini‐Mental State Examination; PFC, prefrontal cortex; SBR, Specific Binding Ratio; SOGS, South Oaks Gambling Screen.

Sixteen out of the 20 subjects with GD (80%) expressed a preference for slot machine gambling (remaining four subjects: horse race betting, bingo, roulette or missing data). None of the GD or control subjects were using medications affecting the dopaminergic system. One individual with GD and one control received 5‐ to 10‐mg/day escitalopram for a mild mood disorder without a diagnosis of major depression; no other selective serotonin reuptake inhibitors (SSRIs) were used by the participants. None had prior or current clinically relevant neurological conditions, and there were no other prior or current psychiatric diagnoses.

### 2.2 Imaging and analysis

SPECT scanning was performed with a Siemens Symbia T6 SPECT/TT system (Siemens Healthineers, Erlangen, Germany). MRI data were acquired with a Siemens 3 T Skyra Fit scanner (Siemens Medical Imaging, Erlangen, Germany) and the imaging protocol included three‐dimensional T1, T2 and FLAIR images. Analyses included both volume‐of‐interest (VOI) based and voxelwise analyses. First, SPECT images were reconstructed with Hermes HybridRecon Neurology (version 2.1.1, Hermes Medical Solutions, Stockholm, Sweden) software including Chang attenuation, LEHR collimator and Monte Carlo‐based scatter corrections. VOI‐based image analyses were then performed using BRASS automated analysis software (version 2.6, Hermes Medical Solutions, Stockholm, Sweden). Specific binding ratios (SBRs) for four regions were calculated (the right and left caudate and right and left putamen) using the occipital cortex as the reference region: SBR = (VOI_caudate or putamen_ − VOI_occipital_)/VOI_occipital_. There were no differences in the occipital cortex (reference region) radioactivity counts between groups (GD mean counts = 10,265, SD = 1383; controls mean = 10,794, SD = 1570; *p* = 0.21).

Voxelwise analyses were conducted using Statistical Parametric Mapping software (SPM12, https://www.fil.ion.ucl.ac.uk/spm/software/spm12/). An average image of the reconstructed and realigned individual scans was calculated and used to estimate a nonlinear transformation to the Montreal Neurological Institute (MNI) standard space by using an in‐house MNI [^123^I]FP‐CIT‐SPECT template.^28^ This transformation was then applied to all individual reconstructed images. The resulting normalization was inspected visually. The occipital cortex was used as the reference tissue to calculate voxelwise SBR images,^29^ which were then smoothed using an 8‐mm Gaussian kernel to improve the signal‐to‐noise ratio. The analysis mask was created using the WFU pickatlas (version 3.0.5, https://www.nitrc.org/projects/wfu_pickatlas/) and included the frontal lobe, cingulate cortex, basal ganglia, thalamus, medial temporal lobe and midbrain. Group differences in regional SBRs were investigated using the general linear model (GLM) with age and sex as covariates. The association between voxelwise SBRs and impulsivity (BIS‐11) was investigated using a GLM with group, age and sex as covariates. This analysis was repeated separately for each impulsivity domain (BIS‐11 nonplanning, attentional and motor subscores). Cluster‐level familywise error (FWE) correction was applied to control for multiple comparisons. FWE‐corrected *P* values less than 0.05 were considered to be significant. The MNI coordinates for up to three peaks at least 8 mm apart were listed for each cluster. Average SBRs were extracted from the significant clusters to illustrate the magnitude and variance of the effects, and for further correlation analyses. In addition, clusters showing a significant group difference were used or correlation analyses with clinical variables.

### 2.3 Statistical analysis

The sample size calculation was done with 1:2 sampling ratio, 80% power and alpha level of 0.05. To detect a 20% difference between group means assuming mean putamen values and SDs from our earlier NMDAT‐project (mean 2.3, SD 0.6), targeted sample sizes were 40 subjects in control group and 20 subjects in GD group. SPSS Statistics (IBM version 27, SPSS Inc., Chicago, IL, USA) was used for statistical analysis of VOI‐based and demographic/clinical data. The assumption of normality was tested with Shapiro–Wilk tests together with histograms. Correlations between variables were calculated with Spearman rank‐order correlation coefficients. For impulsivity, BIS‐11 total score was used and analyses of BIS subscales were considered exploratory. The differences between the groups were calculated using independent samples *t*‐test, Mann–Whitney U test and Fisher's exact test when appropriate. The group difference was also analysed with GLM using BMI, level of education, BDI, motor MDS‐UPDRS and BAI as covariates. The level of statistical significance was set at *p* < 0.05.

## 3 RESULTS

### 3.1 Gambling disorder vs. controls

#### 3.1.1 Demographic and clinical characteristics

In addition to group‐differences in gambling‐ and impulsivity‐related variables, differences between the GD and control groups were observed in BMI (higher in GD), duration of formal education (shorter in GD) and BDI depression scores (higher in GD) (Table 1).

#### 3.1.2 Imaging

**FIGURE 1:**
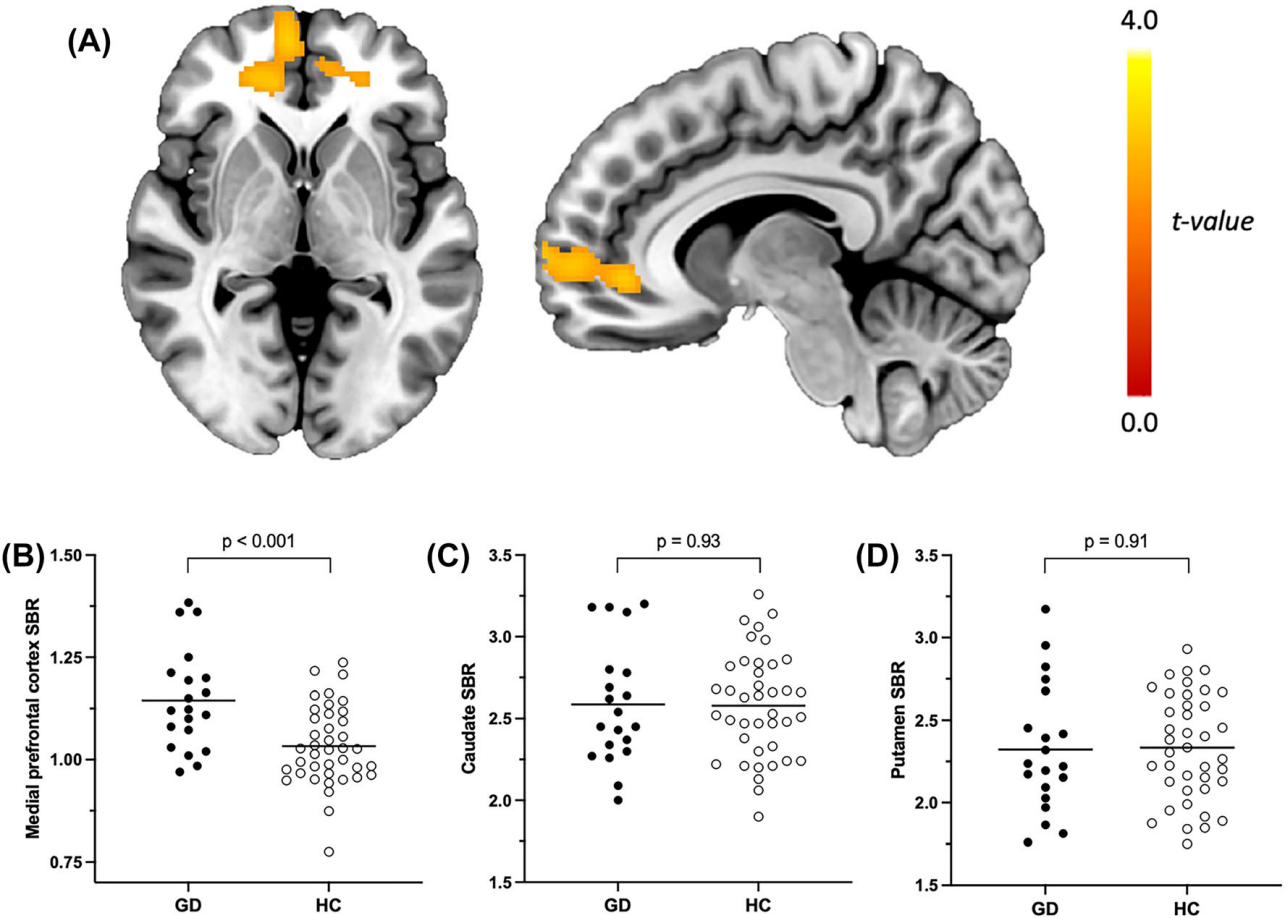
Increased ventromedial prefrontal cortex and normal striatal [^123^I]FP‐CIT uptake in patients with gambling disorder compared to healthy controls. (A) Localization and statistical significance of the cluster in the ventromedial prefrontal cortex. Cluster *P*_FWE_ = 0.04. (B) Individual [^123^I]FP‐CIT specific binding ratio values plotted from the ventromedial prefrontal cortex (B), the mean caudate nucleus (C), and the putamen (D). GD, gambling disorder; HC, healthy controls; SBR, specific binding ratio. Note that cluster values in panel (B) are meant to illustrate the magnitude of the effect observed in the voxewise analysis and not to test any hypotheses.

In the VOI analysis, no differences were observed between GD and controls in caudate nucleus or putamen tracer binding (Table 1 and Figure 1). In the voxelwise analysis, a group difference was observed in the ventromedial prefrontal cortex (vmPFC), with peak voxels at −24 42 26, −26 38 16 and 12 42 −4 (Figure 1). Tracer binding in the vmPFC was 10.7% higher in GD patients than in controls (*p* < 0.001, Table 1). The group difference remained significant in ANCOVA when BMI, level of education, BDI, motor MDS‐UPDRS and BAI were added as covariates (*F* (1, 52) = 4.80, adjusted means 1.11 and 1.05, adjusted group difference 0.06, 95% CI 0.01–0.12, *p* = 0.03). Group differences and correlations remained essentially the same when the two subjects on escitalopram (one GD patient and one controls) were excluded from the analysis.

### 3.2 Correlations with impulsivity and clinical characteristics

#### 3.2.1 Impulsivity

**FIGURE 2:**
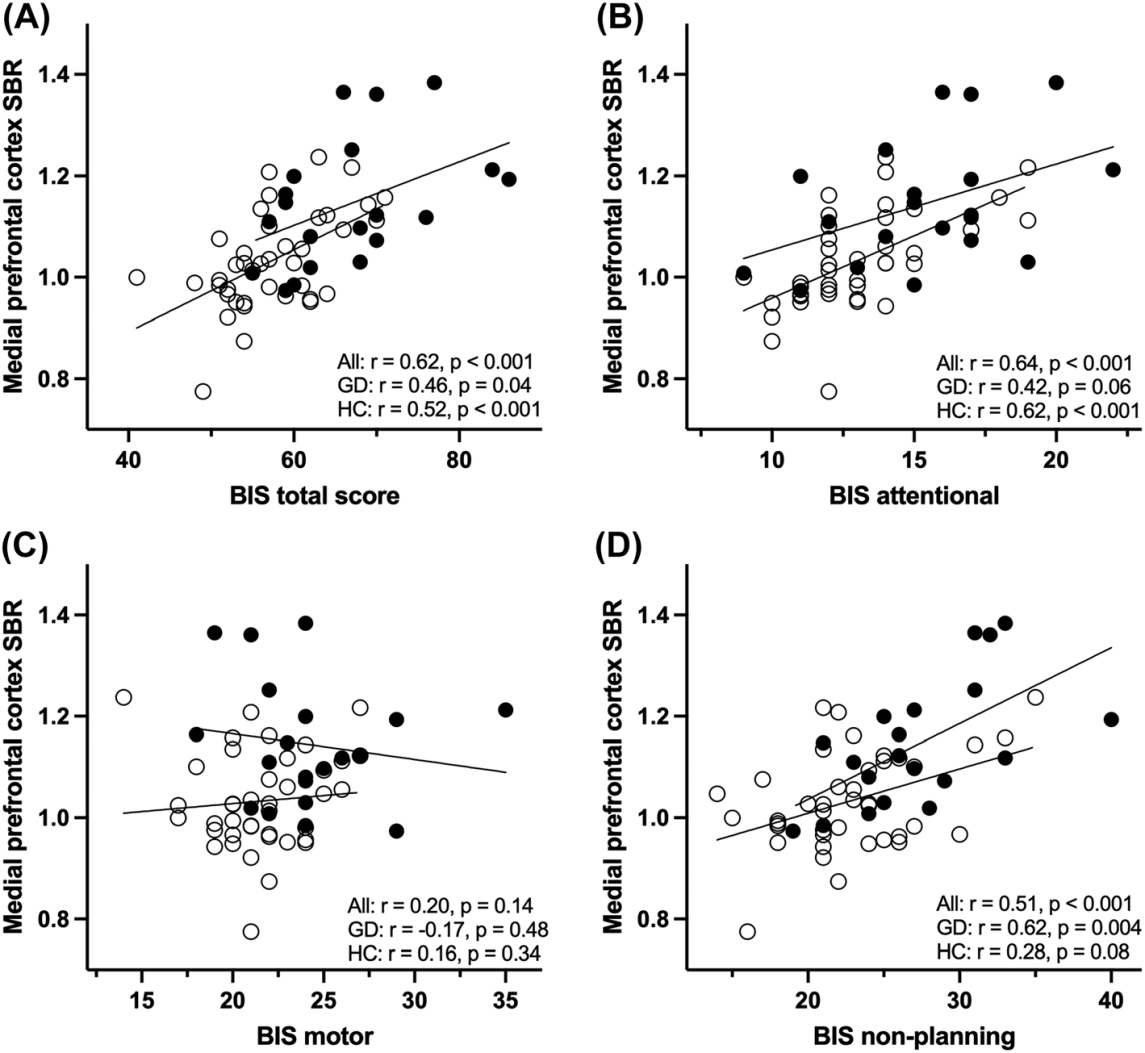
Spearman correlations between ventromedial prefrontal cortex tracer uptake and impulsivity in patients with gambling disorder and healthy controls. (A) Tracer uptake versus Barratt Impulsiveness Scale (BIS‐11) total score. (B) Tracer uptake versus BIS‐11 attentional subscore. (C) Tracer uptake versus BIS‐11 motor subscore. (D) Tracer uptake versus BIS‐11 nonplanning subscore. Solid circles = patients with GD, open circles = healthy controls. GD, gambling disorder; HC, healthy controls; SBR, specific binding ratio

The BIS‐11 total score correlated positively with tracer binding in the vmPFC cluster region in all subjects (GD and controls combined, *r* = 0.62, *p* < 0.001) and separately in healthy controls (*r* = 0.52, *p* < 0.001) and GD patients (*r* = 0.46, *p* = 0.043) (Figure 2). There was no significant difference in the correlation coefficients between healthy controls and GD patients (*p* = 0.79).

#### 3.2.2 Clinical characteristics

**FIGURE 3:**
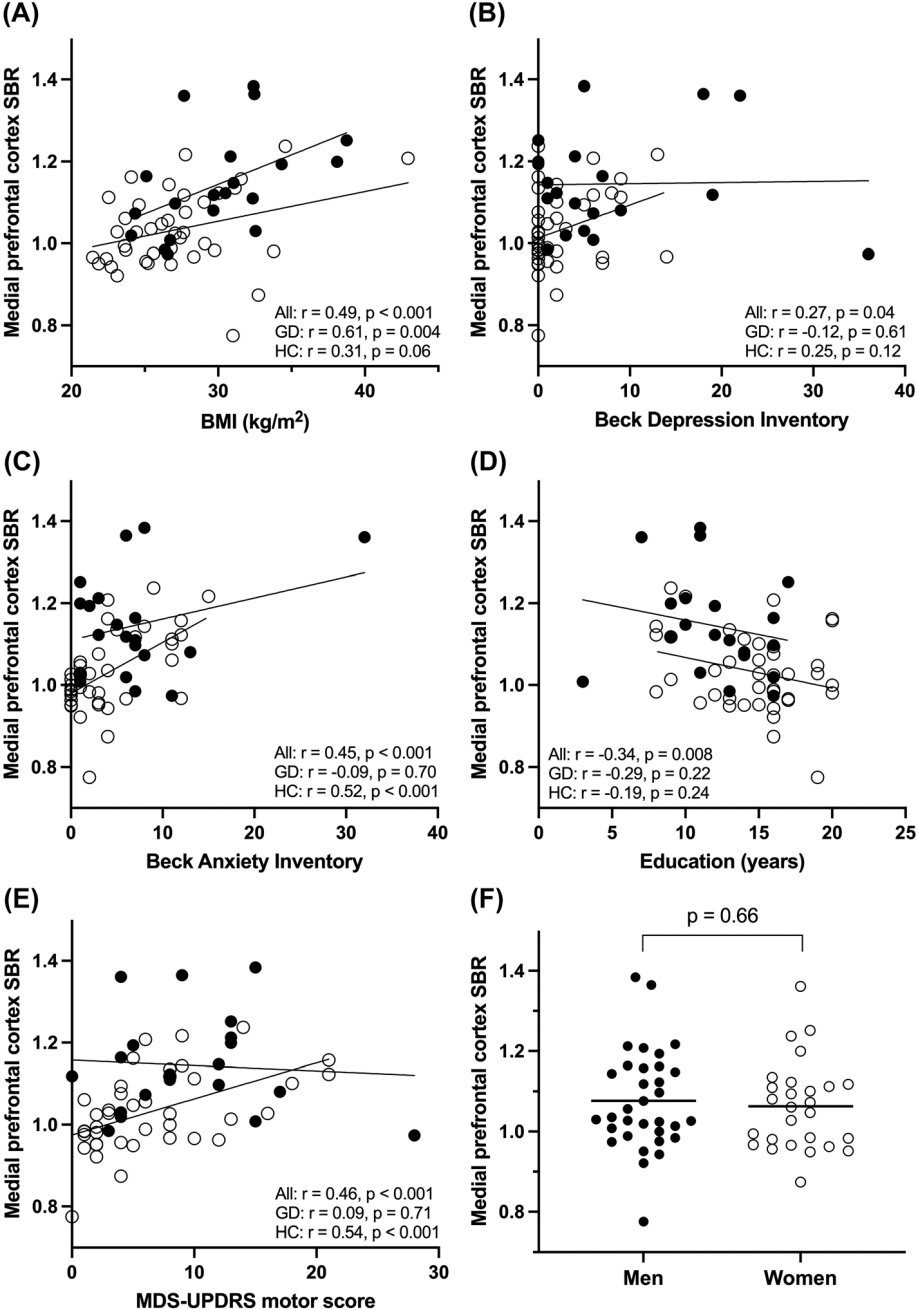
Spearman correlations between ventromedial prefrontal cortex SERT activity and (A) BMI, (B) BDI, (C) BAI, (D) duration of education and (E) MDS‐UPDRS motor score. No sex‐differences were observed in binding (F). BMI, body mass index; SBR, specific binding ratio

Striatal or vmPFC tracer binding did not correlate with SOGS scores (*r* < 0.21, *p* > 0.39). Age did not correlate with striatal or vmPFC binding in healthy controls (*r* > −0.27, *p* > 0.09) or GD patients (*r* < 0.19, *p* > 0.41). Tracer binding in the vmPFC correlated with BMI (*r* = 0.61, *p* = 0.004) in GD patients and with BAI (*r* = 0.52, *p* < 0.001) and motor MDS‐UPDRS (*r* = 0.54, *p* < 0.001) in healthy controls, but other clinical characteristics did not correlate with vmPFC tracer binding in GD patients or healthy controls (Figure 3).

#### 3.2.3 Subscales of impulsivity

**FIGURE 4:**
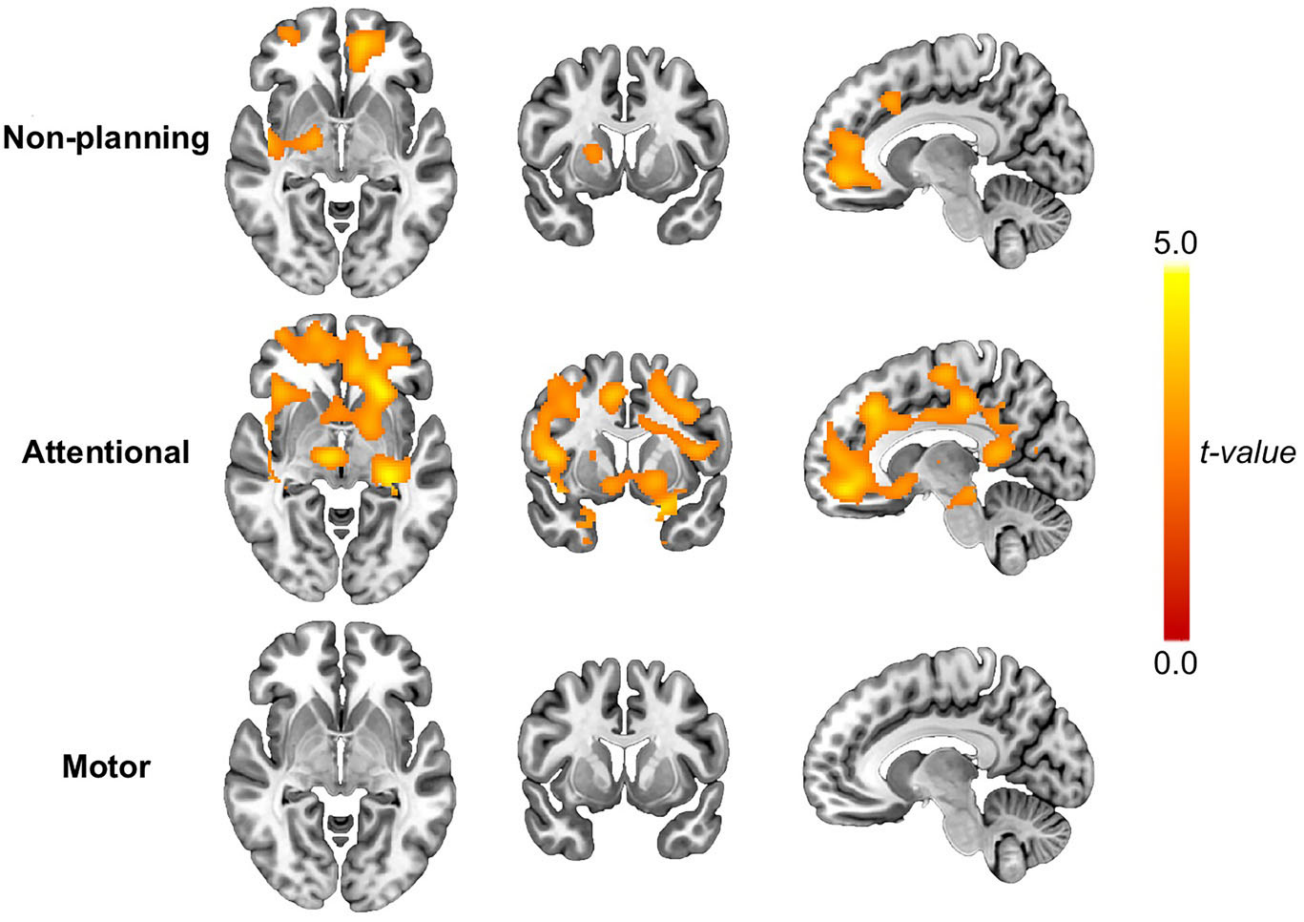
Association between impulsivity and [^123^I]FP‐CIT uptake. Clusters of voxels with a positive association between [^123^I]FP‐CIT uptake and nonplanning (upper row), attentional (middle row) and motor (bottom row) impulsivity. *P*_FWE_ < 0.05 for all clusters. There was no significant group x impulsivity interaction.

We conducted exploratory analyses investigating subfactors of impulsivity. Positive correlations were observed between vmPFC binding and BIS‐11 attentional and nonplanning subscores but not with the BIS‐11 motor subscore (Figure 2). Over the whole brain and all subjects, nonplanning and attentional impulsivity were significantly associated with tracer binding whereas motor impulsivity was not (Figure 4). Nonplanning impulsivity was associated with tracer binding in the prefrontal cortex and anterior cingulate (peak voxels at Montreal Neurological Institute *x*,*y*,*z* coordinates in mm: −20 48 14, 20 38 20, 34 38 12), and the putamen, pallidum, posterior insula and medial temporal lobe (peak voxels at 44 −8 −8, 38 −18 32, 18 2 8) (Figure 4, first row). Attentional impulsivity was associated with tracer binding in widespread areas covering the entire cingulate, ventral striatum, and regions in the insula and prefrontal cortex, and regions in the midbrain (peak voxels at −34 −22 −18, 4 −4 38, −16, 30, 36) (Figure 4, second row). Clusters associated with nonplanning and attentional impulsivity overlapped with the observed group difference.

## 4 DISCUSSION

We highlight aberrant prefrontal serotonergic and striatal dopaminergic monoaminergic neurotransmission in older subjects with GD. First, we showed that extrastriatal [^123^I]FP‐CIT binding, a putative marker of SERT, was increased in the vmPFC of individuals with GD compared to subjects without gambling problems. Second, this marker of SERT function in the vmPFC correlated with impulsivity in GD patients and healthy controls providing a relevant link to the phenotype of GD. Third, striatal DAT binding was associated with impulsivity, but no group differences were observed in striatal DAT function in GD pointing to a contrast between dopaminergic consequences of substance use disorders^30^, ^31^, ^32^ and GD, a behavioural addiction without chronic pharmacological effects of drugs.

The increased uptake of [^123^I]FP‐CIT in the vmPFC of GD patients likely reflects greater SERT function. The tracer has affinity for both presynaptic DAT^23^ and SERT,^24^ which enables its use as a proxy for DAT in the striatum but for SERT extrastriatally.^25^ Prefrontal cortical SERT‐expressing neurons appear to have an important role in the circuitry from the prefrontal cortex to the dorsal raphe nucleus,^33^ and the medial prefrontal cortex has previously been implicated in multiple aspects of drug abuse and addiction, including drug‐seeking motivation and craving.^34^ Specific deficits in medial prefrontal processing have been reported to lead to impaired decisions that involve risk and in rats, the activity of prefrontal neurons predicts future choices during a gambling task.^35^, ^36^ It is noteworthy that our earlier SERT study with [^11^C]MADAM positron emission tomography (PET) found no differences in tracer binding between GD patients and healthy individuals,^18^ although the number of subjects in our earlier study was considerably lower (*n* = 30 vs. *n* = 60 in the present study) and subjects were younger. We note that the present results remained significant after FWE‐correction for multiple comparisons and are in line with our [^18^F]fluorodopa PET study, focusing on impulse control disorders (ICDs) including GD in the context of Parkinson's disease (PD).^37^ The [^18^F]fluorodopa uptake, reflecting striatal dopaminergic activity and extrastriatal monoaminergic activity, was higher in the medial prefrontal cortex of PD patients with ICDs than in those without ICDs. The combined results thus point to a higher medial prefrontal monoaminergic, probably serotonergic, activity in individuals with ICDs and GD.

The second main finding of this study was the positive relationship between vmPFC tracer binding and impulsivity, over the whole sample and separately in GD and controls. In the whole sample and in controls, the statistical significance was high (*p* < 0.001). Impulsivity, referring to premature, unduly risky, poorly conceived actions, is strongly associated with GD as well as substance use disorders.^20^, ^22^, ^38^, ^39^ Data from a number of earlier studies suggest an important role of the prefrontal cortex in controlling multiple types of impulsivity^40^ with the vmPFC, particularly implicating delay discounting, risk taking and goal‐directed control.^41^ Rodent models have identified enhanced SERT function in the orbitofrontal cortex and medial prefrontal regions as a potential mechanism that underlies impulsive behaviour contributing to reward processing.^42^, ^43^ The literature linking serotonergic transmission to ‘waiting’ impulsivity or premature responding is extensive but mostly based on animal studies. ‘Waiting’ impulsivity in humans implicates the vmPFC or human analogue of the prelimbic cortex^44^ and tryptophan depletion lowering central serotonergic levels in healthy humans also results in greater ‘waiting’ impulsivity.^45^ Genetic or pharmacological disruption of SERT or administration of agents that elevate synaptic 5‐HT levels reduce premature responses in animals, whereas depletion of brain 5‐HT has the opposite effect.^46^ In humans, tryptophan depletion studies have indicated an inverse relationship between brain serotonin levels and behavioural impulsivity.^47^ Our observation of the link between increased SERT binding and increased impulsivity in human GD patients parallels previous findings in animals and humans, since the result could be a reflection of compensatory activation of SERT in response to depleted 5‐HT levels in the vmPFC of GD patients. Together, our findings support the conceptual premise that the vmPFC and SERT may be potential treatment targets in GD. SSRIs were one of the first medications tested for treating GD, and although fluvoxamine, paroxetine and escitalopram showed some efficacy in GD, there were also negative results, and controversy remains regarding whether SSRIs can reduce urges to gamble or whether they rather treat associated anxiety and depressive symptoms in GD.^48^ Another therapeutic possibility is noninvasive neuromodulation, which has shown some preliminary benefit in GD^49^ and could be targeted to a specific cortical regions, such as the vmPFC. A related example is the efficacy of high‐frequency transcranial magnetic stimulation (TMS) in nicotine addiction,^50^ which was recently approved by the US Food and Drug Administration for smoking cessation in adults. TMS has also shown some promise in modulating striatal DAT function in GD.^51^

Third, we found no evidence for alterations in DAT binding in GD compared to controls, although striatal DAT binding was also positively correlated with impulsivity scores in both groups, which is in line with a previous [^123^I]FP‐CIT SPECT study in healthy subjects,^52^ although an opposite correlation^53^ and negative results^54^ have also been reported. One earlier study reported an 11%–13% striatal DAT binding decrease in GD compared to controls.^16^ The reasons for differences in the results between the earlier smaller study (GD *n* = 15 vs. controls *n* = 17)^16^ and the present study are not clear. However, in the earlier study, all but one subject were males, subjects were considerably younger and impulsivity scores were somewhat higher. The dopamine system shows substantial age‐related changes (e.g., in D2/3 binding), but older age is increasingly recognized as a vulnerable window for disordered gambling.^55^ In addition, some work has indicated nonlinear (i.e., U‐shaped) relationships between dopamine D2/3 receptor binding and impulsivity, both in GD and healthy participants, such that both high and low extremes of impulsivity may be associated with reduced dopamine transmission.^9^, ^56^ It is possible that these factors explain the differences compared to the present study, although we did not observe reduced DAT binding in our male GD patients compared to male controls (data not shown) and the group difference in striatal binding values was clearly nonsignificant in our study without any trend‐level findings. The mean age of our sample underlines the issue of lack of striatal differences and higher prefrontal binding. First, the results are unlikely to be related to age‐/GD‐associated brain atrophy, since atrophy would have induced partial volume effects and would have led to lower binding ratios in GD. Second, there are prior reports that have linked substance use disorders with acceleration of normal ageing, possibly driven by immunosenescence and accelerated telomere shortening.^57^, ^58^, ^59^ If this theory applies to GD, one would expect that the degeneration of DAT and SERT systems would be particularly prominent in aged individuals with long‐term GD. Instead, transporter function was normal (DAT), arguing against accelerated cellular senescence in GD. It is also important to note that although we did not observe an association between the dysfunction in neurotransmission and the duration of GD behaviour, the transition time between problematic gaming and pathological gambling may be longer in older individuals with GD.^60^ It is therefore possible that neural adaptation and compensatory mechanisms are more involved in older patients, and in the interpretation of the present findings, it should be noted that most GD patients in clinical setting are younger and have more psychiatric comorbidities than the present targeted sample of individuals with GD. In relation to older patients, multiple studies of dopaminergic medication‐related behavioural addictions in Parkinson's disease demonstrate lower dopamine transporter levels in the striatum, highlighting differences in the disorders that are likely related to the relationship with dopaminergic medications or Parkinsonian pathology.^61^ Finally, genetic susceptibility may also be important, as has been hypothesized in aripiprazole‐induced GD via genetic polymorphism on the dopamine D2 receptor.^62^

To summarize, we have demonstrated increased prefrontal SERT function in GD associated with individual differences in impulsivity. Although we focused on GD, the findings may have a bearing on the understanding of neurotransmitter function in other behavioural and substance addictions and particularly their relationship with impulsive behaviour. An implication of this is the possibility that pathologically increased impulsive behaviour may be modulated in humans by interventions that target 5‐HT function in the vmPFC. Thus, although the results should be replicated independently with a larger number of younger GD patients, our findings highlight potential novel targets for neuromodulation both for GD and the dimension of impulsivity.

## CONFLICT OF INTEREST

There are no relevant conflicts of interest.

## AUTHOR CONTRIBUTION

VK was responsible for the study concept and design. VK, EAH, KL, EJ, JM, RP, TN, JJ and MP contributed to the acquisition of data. VK, TV, VV, LC and JJ assisted with data analysis and interpretation of findings. VK drafted the manuscript. VV, LC and JJ provided critical revision of the manuscript for important intellectual content. All authors critically reviewed content and approved final version for publication.

## Data Availability

The data that support the findings of this study are available from the corresponding author upon reasonable request.
